# Enhanced oxidative stability of meat by including tannin-rich leaves of woody plants in goat diet

**DOI:** 10.5713/ajas.18.0537

**Published:** 2019-01-03

**Authors:** Elisa Mariana García, Agustín López, María Zimerman, Olegario Hernández, José Ignacio Arroquy, Mónica Azucena Nazareno

**Affiliations:** 1 Laboratory of Antioxidants and Oxidative Processes, Institute of Chemical Sciences, Faculty of Agronomy and Agroindustries, National University of Santiago del Estero, El Zanjón, Santiago del Estero, 4206, Argentina; 2 National Scientific and Technical Research Council-Argentina, Buenos Aires, C1425FQB, Argentina; 3 Laboratory of Forages and Animal Nutrition, Experimental Agropecuary Station, National Institute of Agricultural Technology, La Abrita, Santiago del Estero, 4206, Argentina; 4 Semi-arid Chaco Animal Research Institute, Agricultural Research Center, National Institute of Agricultural Technology, Leales, Tucumán, 4113, Argentina

**Keywords:** Meat Quality, Meat Oxidation, Larrea Divaricata, Acacia Aroma, Phytochemicals, Bioactive Compounds

## Abstract

**Objective:**

The aim of this study was to evaluate the effect of dietary incorporation of tannin-rich woody species on meat oxidative stability, carcass traits and meat quality in goats.

**Methods:**

Two tannin-rich species were tested using a three-treatments feeding trial, where treatments consisted of: *Larrea divaricata* and *Acacia aroma* both at 12.5% in dry matter basis of the diet and a control diet (alfalfa hay). All feeding diets were iso-protein and iso-energy. Carcass conformation, carcass compactness, carcass fatness and subcutaneous fat deposition were evaluated. Intake, liveweigh, *Longissimus thoracis et lumborum* muscles of goats were analyzed in order to evaluate quality parameters such as pH value, instrumental color evaluation, water holding capacity, total phenolic content, antioxidant activity, meat oxidative stability and fatty acid profiles in meat.

**Results:**

Feed intake, liveweight gain, carcass, and meat traits did not differ among treatments. Changes in meat lipid profile among treatments were observed for oleic and elaidic acid contents. Meat total phenolic content and antioxidant activity did not differ among treatments; although, meat oxidative status after storage at room temperature, as well as under refrigerated and frozen conditions were different between control and both supplemented groups.

**Conclusion:**

The inclusion of *Acacia aroma* and *Larrea divaricata* leaves in goat diet enhanced meat oxidative stability. Modulation of the ruminal biohydrogenation of fatty acids produced by condensed tannins of these plant species need to be further investigated.

## INTRODUCTION

Goat meat is considered a healthy product and represents an important source of animal protein and iron for human diet [1]. However, oxidation of lipid fraction is considered one of the main non-microbial causes affecting meat quality. The rate and degree of meat oxidation can be delayed or avoided by the application of antioxidants [2].

In food industry, a regular strategy to prevent or retard food’s oxidation implies the addi tion of antioxidants directly to food formulation or coated containers made with antioxidant agents [3,4]. On the other hand, further strategies of protection against meat oxidative damage involve the increase of intrinsic antioxidant levels by incorporating them through animal diets. Furthermore, the inclusion of natural antioxidants in the animals’ diet could reduce meat oxidation rate but also improve its quality. For instance, the dietary inclusion of vitamin E enhanced the oxidative stability of beef meat [5].

In goats’ production systems of the semiarid Argentine Chaco, woody perennial plants represent an important source of supplementary feed during the dry season when fresh forage availability is limited [6]. Woody plants produce a great variety of bioactive compounds such as condensed tannins (CT). The CT were considered in the past as anti-nutritive and toxic compounds due to their adverse nutritional effects for herbivores, especially when they are present in high concentration in plant tissues [7,8]. Nevertheless, tannin-rich plants might improve nutrient utilization by modifying feed efficiency and/or nutritional qualities of animal products (e.g. meat and milk) when ingested in moderate levels. Besides, CT may have beneficial effects on ruminants by preventing bloat, increasing digestive utilization of dietary protein, and acting as anthelmintic and as antioxidants [9,10]. In previous research [11], it was observed that CT from *Acacia cyanophylla*, *Schinopsis lorentzii*, and *Ceratonia siliqua* reduced the biohydrogenation of polyunsaturated fatty acids (PUFA). On the other hand, supplementing the goat diet with pine bark rich in CT did not find differences in meat oxidative stability against a diet with low tannins input [12]. In a preceding study, it was shown that there were differences in nutritive value and bioactivity, measured in terms of the protein precipitation capacity, among native woody species of the Argentine Dry Chaco [13]. Some species high in CT concentration were not necessarily those with the highest biological activity. *Caesalpinia paraguariensis*, *Schinopsis balansae*, and *Larrea divaricata* (LD) were the richest species in CT as native forage. However, LD and *Acacia aroma* (AA) had the highest biological activity, followed by *Prosopis alba*. According to their nutritive traits, these species might be also complementary in grass-based ruminant diets [13].

Based on the above, the main objective of this work was to evaluate the effect of the inclusion of two native woody species (LD and AA) as a source of CT in the diet for goats, on animal performance, feed intake, carcass traits and meat quality as well as on meat oxidative status and oxidative stability during refrigerated storage.

## MATERIALS AND METHODS

### Collection of feeding material

Leaves of LD and AA were collected from native wild plants in the Argentine Dry Chaco (27°47′3.91″S, 64°16′2.21″W) in November 2014. Leaves were harvested and air-dried under shade on a clean plastic. Once leaves were dried, they were easily removed from the branches and bagged until their use. LD and AA leaves were characterized according to their secondary metabolite composition as indicated by García et al [13], being their total phenolic contents (TPCs) 13.8±0.5 and 29±2 mg gallic acid equivalents/100 g of dry matter (mg GAE/100 g DM), and their CT contents 1.39 and 4.50 g/100 g DM, respectively.

### Animals and diet

Nineteen goats (*Capra hircus* “Creole” breed; 4 mo. old and initial average liveweight: 10.2±0.9 kg) were randomly assigned to three experimental diets (Table 1): Control (n = 7) an alfalfa-corn-soybean meal diet without tannin-rich leaves; while, LD (n = 6) and AA (n = 6) with the same basal diet but with 12.5% in replacement of alfalfa hay by the native species. Experimental diets were iso-protein and iso-energy. The energy and protein contents were estimated based on the Creole goat requirements and adjusted to the same values for all the treatments; basal diet was formulated according to the NRC [14]. Prior to the beginning of the experiment, the goats were adapted to the basal diet for 10 d, then, they were fed 50 d for the experimental period. The goats were fed individually with the corresponding diet for each treatment every morning at 0900 h. Goat have free water access during the study. The composition of the experimental diets is described in Table 1. Feed refusal were collected every day to do a weekly composite sample per animal, in order to estimate the daily voluntary feed intake.

### Slaughter procedure, carcass traits, and meat sampling

The feeding trial was conducted at the Experimental Station INTA (Instituto Nacional de Tecnología Agropecuaria) in Santiago del Estero, Argentina. Animal handling and experimental procedures were conducted in accordance with regulation procedures for animal welfare of the National Service of Animal Health (Servicio Nacional de Sanidad y Calidad Agroalimentaria, SENASA) of Argentina. Animals were slaughtered at 180 d old at an experimental abattoir. Goats were deprived of food 24 h before slaughter; although, they had free access to water. Slaughter live weight (SLW) was determined immediately before slaughter. Kid goats were slaughtered by exsanguination. The dressed carcass comprised the body after removing skin, head (at occipital-atlantal joint), fore feet (at carpal-metacarpal joint), hind feet (at tarsal-metatarsal joint) and viscera. Tail, diaphragm, kidneys, and fat around the kidneys and pelvic area were kept in the carcass. Hot carcass weight was recorded immediately after slaughter while cold carcass weight (CCW) was measured after 24 h, at 4°C. Dressing percentage was calculated as the percental relation between CCW and SLW (SLW = CCW/LW×100). Objective carcass conformation was measured according to the methodologies recommended by Bonvillani et al [15], taking into account internal carcass length, hind limb length, buttock perimeter, buttock width, thorax depth, thorax width leg compactness (leg weight/hind limb length; kg/m) and carcass compactness (CCW/internal carcass length; kg/m). Carcass fatness was evaluated according to the kidney surface covered with fat, comparing carcass with photographic models of a five-point scale (0, 0.25, 0.5, 0.75, and 1) as proposed by Domingo et al [16]. In addition, subcutaneous fat deposition was evaluated in the same way by comparison with a photographic scale (1, very lean; 2, lean; 3, medium fat; 4, fat; and 5, very fat) according to methodology proposed by Colomer-Rocher et al [17,18]. Subsequently, the *Longissimus thoracis et lumborum* muscle was removed from carcass and refrigerated at 4°C. Meat sample was divided into different portions: one (*Longissimus thoracis* portion) was processed 24 h after slaughter to evaluate quality parameters and the other one (*Longissimus lumborum* portion) was vacuum-packed and aged for additional two days at 2°C±1°C and, subsequently, processed to evaluate oxidative stability.

### Meat measurements

Meat pH and temperature were measured at 24 h post-slaughter between the 4th and 5th lumbar vertebra with a portable pH meter (PHS3D pH Meter Model PHS-3D-02 Assembly SANXIN, Shanghai San-Xin Instrumentation, Inc., Shanghai, China), calibration was performed using buffer pH 4 and 7. Instrumental color (L*, a*, and b*) was measured as reported in Luciano et al [19], using Minolta l CR300 colorimeter (Konica, Minolta Camera Co. Ltd., Osaka, Japan). Measurements were made from a 0° viewing angle at 8 mm aperture and using illuminant C. The determinations were performed on cross-section at the region of the 1st lumbar vertebra. Meat samples were taken at 24 h after slaughter after 30 minutes of oxygenation or “blooming” time at 2°C±1°C. Triplicate readings were made on non-overlapping zones of the sample and average values were calculated. Chroma (C*) was calculated as (a*^2^+ b*^2^)^1/2^ and hue angle (H*) as tan^−1^(b*/a*)×(180/**π**). Water holding capacity (WHC) was determined by duplicate according to the compression method described by Irie et al [20].

Meat samples from *Longissimus lumborum* left portion were processed to measure TPC and antioxidant activity. Extracts were prepared homogenizing 5 g of meat in 10 mL of 0.05 M phosphate buffer (pH 7). Mixture was centrifuged at 18,000×*g* for 20 min at 4°C.

The TPC was determined by Folin-Ciocalteu method as suggested by Singleton and Rossi [21] as follows: 0.2 mL supernatant was added to 0.65 mL of 1 N Folin-Ciocalteu reagent, followed by the addition of 3.15 mL 5% sodium carbonate solution and 1 mL of ultra-pure water. After incubation at 50°C for 15 min, reaction mixture was vortexed. Phenolic content was determined by measuring the absorbance using UNICAM UV2 equipment (UNICAM Limited, Thermo Optek, Torfaen, UK) at 725 nm. Gallic acid (GA) was used for calibration and TPC was expressed as GAE/g of meat. The radical-scavenging activity against 2,2-diphenyl-1-picrylhydrazyl radical (DPPH·) was determined according the method suggested by Brand-Williams et al [22]. An aliquot of 2 mL of 0.2 mM DPPH· radical solution prepared in methanol was added to 200 μL supernatant and 800 μL methanol. The mixture was vortexed and left to stand at room temperature for 10 min. Absorbance was measured at 517 nm. The scavenging activity of meat sample against DPPH· radical was expressed as mg GAE/g of meat.

The oxidative stability in meat samples was performed mea suring 2-thiobarbituric acid reactive substances (TBARS) levels after different storage conditions.

After the ageing process (3 d), meat samples from the *Longissimus lumborum* right portion were divided into three fractions. One of them was stored at 26°C for six hours, and the remaining two samples were both vacuum packaged, being the second one stored at −18°C for 30 d and the third one at 4°C, respectively, for 0, 2, 4, and 6 d. Each sample was placed in an individual plastic bag.

Lipid oxidation was assessed according to the method described by Botsoglou et al [23]. Meat samples (1 g) were homogenized with 3 mL of distilled water using a Bio-Gen Series PRO 200 Pro Scientific tissue homogenizer at 9,500 rpm for 45 s. Subsequently, 0.75 mL of 25% (w/v) trichloroacetic acid was added to precipitate proteins and then, mixture was stirred for 15 minutes at 4°C. The homogenates were centrifuged at 18,000×*g* for 15 min at 4°C. An aliquot of 1 mL of supernatant was added to 2 mL de 0.6% (w/v) aqueous thiobarbituric acid solution into screw cap glass tubes. Tubes were incubated in water bath at 70°C for 30 min, and absorbance was read at 532 nm. The assay was calibrated with solution of known concentration of 1,1,3,3-tetraethoxypropene in distilled water. Results were expressed such as mg of malondialdehyde (MDA)/g of meat.

Determination of fatty acids profile after derivatization as methyl esters was performed by gas chromatography with flame ionization detector (GC-FID). Intramuscular lipid from muscle sample was quantitatively extracted, according to the method of Bligh and Dyer [24]. The extraction was done by combination of three solvents as ternary system: chloroform, methanol and water. This procedure was divided into two stages; the extraction with a (20:35:45) water- chloroform-methanol mixture. In the second stage, a (32:40:22) water-chloroform-methanol mixture was used. Organic phase was separated and a rotary evaporator was used to eliminate solvent under vacuum from the fat extract at 40°C. Before the extraction, 2-tertbutyl-4-hydroxyanisole (BHA) was added at 0.01% as antioxidant. The fat extracted from the muscle was stored in a glass vial sealed under nitrogen and frozen at −20°C until determination. Approximately 30 mg of extracted fat was saponified with 4 mL of 0.5 N methanolic NaOH for 2 min/90°C. After cooling, an aliquot of 5 mL of 14% boron trifluoride in methanol was added and heated for 2 min at the same temperature as above. Then, reaction tubes were cooled; 4 mL of n-hexane and 10 mL of saturated NaCl solution were added and mixed for 30 s and then, solution was let until organic and aqueous phases separated. Fatty acid methyl esters (FAME) were collected from the top layer, transferred to a vial and stored at −20°C until GC-FID analysis. FAME were quantified using a gas chromatograph (HRGC-3000C; Konik Group, Barcelona, Spain) equipped with a fused silica capillary column HP-88 (60 m length, 0.25 mm internal diameter, 0.20 μm film thicknesses); Agilent Technology Inc. (Santa Clara, CA, USA). Analysis was performed using an initial isothermal period (90°C for 5 min); thereafter, temperature was increased to 230°C at a rate of 7°C/min and kept at this temperature for 2 min. Then, temperature was increased to 250°C at a rate of 2°C/min; finally, an isothermal period of 250°C was kept for 30 min.

The FAME constituents in the sample were identified by comparing the retention times of sample peaks with those of standards obtained from Supelco (Supelco 37 Component Fame Mix 47885-U, Sigma Aldrich, Bellefonte, PA, USA). Analytic results were expressed as percentages of total fatty acids.

### Statistical analysis

All data were analyzed as a completely randomized design with the linear mixed-effects model procedure through the R interface, using INFOSTAT 2014 Statistical Software (UNC, Argentina) [25] for variables DPPH· bleaching activity, TPCs, meat pH and color parameters. The model includes the fixed effects of diet and random effects of experimental unit (animal). The model used for analysis of variance was:

$${\text{Y}}_{\text{ij}}=\mu +\text{Di}+\text{Aj}+ɛ\text{ij}$$

Where Yij is the response to diet, μ is the overall mean, Di is the fixed effect of diet, Aj is the random effect of animal and ɛij is the experimental error.

Data related to lipid oxidation (TBARS values) were ana lyzed as repeated measures. The model for repeated measures analysis was:

Yijk=μ+Di+Aj+tk+(Di×tk)+ɛijk

Where Yijk is the dependent variable, μ is the global media, Di is fixed effect of diet i, Aj is random effect of animal k, tk fixed effect of time k, (Di×tk) is fixed effect of the interaction between treatment j and time k, and ɛijk is experimental error. Multiple comparisons among means was done using LSD Fisher test (p<0.05).

## RESULTS AND DISCUSSION

### Slaughter and carcass characteristics

There were no significant differences in average daily gain neither on final live weight nor hot carcass percentage among treatments (Table 2). Min et al [26] suggested that feeding CT-containing plants increases rumen bypass proteins, decreasing the protein levels available for rumen digestion. The present study did not reveal the increase in bypass protein since similar performances were obtained between treatments. Consistenly, no significant differences were found in hot carcass (Table 2).

Carcass measurements and compactness (Table 2) were not affected by treatments (p>0.05). Futhermore, carcass fatness, measured by kidney fat cover was also not affected by treatments (p>0.05).

### Meat evaluation: pH values, instrumental color, and water holding capacity

The final pH value is a relevant parameter due to its effect on shelf life, color and quality of fresh meat. In the present study, ultimate pH did not differ among treatments (Table 3). Similar results were obtained by other authors. Lee et al [12] found that meat ultimate pH values were not affected by CT inclusion in the diet of goats; meat ultimate pH from those goats fed with pine bark (13% CT) was 5.70; while, that from goats fed with bermudagrass hay (0.12% CT) was 5.65. Furthermore, Francisco et al [27] reported that meat pH values in lambs treated with diets containing different CT levels (CT % from 2.5 up to 16.3) were not different each other.

The visual appearance of fresh meat is based on color and WHC [28]. Along with the amount of fat, meat color is of great importance because these two characteristics will be the first to determine consumer purchasing decisions [29]. Changes in a* (redness) and b* (yellowness) values over a period of time describe meat color deterioration from red to brown, and reflect the myoglobin concentration and its redox state in meat [30].

In the present study, no differences were found in meat color due to nutritional treatment (Table 3). The lightness values, L*, on average were 56 and the values of the coordinates a* and b* were 9.19 and 1.88 respectively. The a* values decreased with the increase of polyphenols in the diet, the values for the control group, LD and AA were 9.5, 9.24, and 8.83 respectively. Likewise, it was also observed that the b* values increased according to the polyphenol contents of the diet giving values of 1.55 for the control group, 2.04 for the group supplemented with LD and 2.06 for the group supplemented with AA. The trend observed for redness and saturation is in agreement with the results found by Inserra et al [31], who reported a reduction in a* and Chroma values of lamb meat consequent to the dietary inclusion of dried citrus pulp in a concentrate containing barley. In the same way, Lee et al [12] studied the supplementation of goats with bermudagrass and pine bark, and found lower a* values in supplemented animals compared to those fed only with bermudagrass and reported no differences on L* and b* values. Finally, Luciano et al [19] contrasted two diets in lambs: concentrate and inclusion of quebracho tannins on food. The authors found an increase in a* values and a reduction in b* ones on animals supplemented with tannins in diet.

### Phenolic compounds and antioxidant activity of meat

Polyphenols are natural antioxidants that have the ability to reduce oxidative damage, exerting a natural protective action on cell against reactive oxygen species, either trapping free radicals or inhibiting their action. For this reason, in recent years, plants and vegetal extracts rich in polyphenolic compounds have been extensively studied as possible sources of natural antioxidants in animal nutrition. Total phenol contents of meat varied from 10.7 to 11.1 mg GAE/g meat without significant difference among treatments. Although, there were not statistical differences, meat of goats supplemented with AA had numerically the highest content of phenolic compounds followed by those fed with LD.

In the same trend, our data did not reveal significant vari ations in the meat antioxidant activity due to the supplemented diets of goats (Table 3).

### Lipid oxidation of meat during refrigerated storage

**Figure 1:**
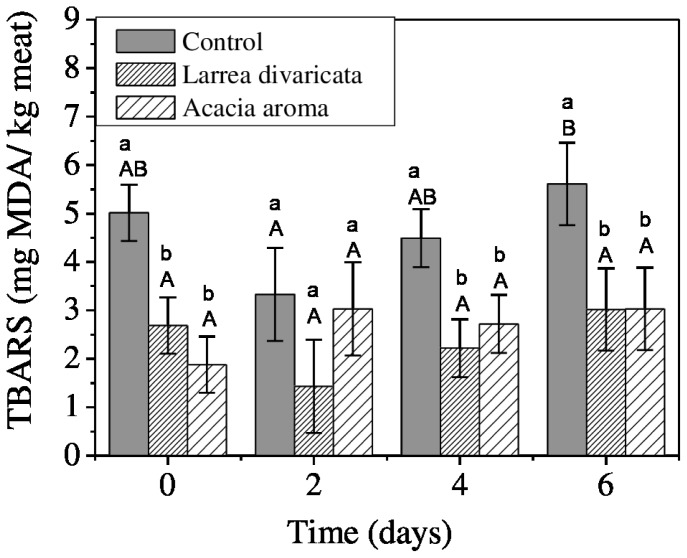
Main effects of dietary treatment and storage time at 4°C on meat oxidative status measured in *Longissimus lumborum* muscle. Values presented are the mean 2-thiobarbituric acid reactive substances values and standard error bars. ^a,b^ Within each day ofstorage, different superscripts indicate differences between dietary treatments and ^A,B^ Within each dietary treatment, different superscripts indicate differences among storage days, using LSD Fisher test adjustment for multiple comparisons (p<0.05).

The effect of different diets on TBARS values in refrigerated meat at 4°C for 6 days are shown in Figure 1. During the first day of storage, TBARS value was significantly (p = 0.003) higher in control group than AA and LD, value for meat supplemented with AA being 1.88 mg MDA/kg of meat, followed by LD, 2.69 mg MDA/kg of meat. Finally, the meat that presented the highest state of oxidation was that of the control group, 5.01 mg MDA/kg of meat.

Lipid oxidation was affected by the storage conditions, a significative increase in TBARS along storage period was observed in control group with respect to LD and AA groups. The results obtained for the TBARS values were 2.22 and 3.02 mg MDA/kg of meat for goats supplemented with LD and 2.72 and 3.03 mg MDA/kg of meat for those supplemented with AA at the fourth and sixth days of storage, respectively. The largest increase, up to 5.61 mg MDA/kg of meat after six days of storage, was observed in the control group. Only at the second day of storage was no difference in TBARS accumulation observed among experimental groups. Moreover, it is noteworthy that, meat TBARS of LD and AA groups were around the threshold value suggested as a sensorial detection of meat rancid flavors proposed by Campo et al [32], which is 2 mg MDA/kg of meat, during nine days of storage.

**Figure 2:**
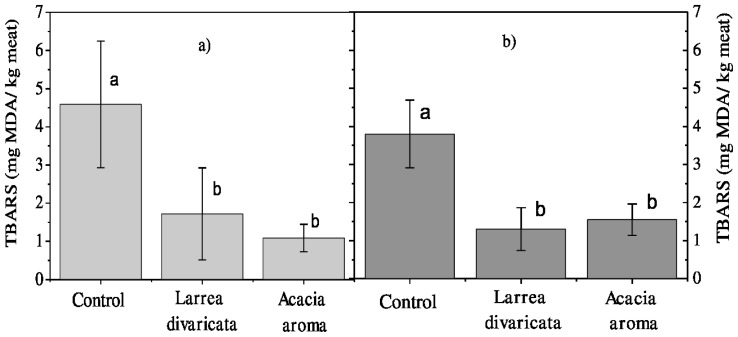
Main effects of dietary treatment on oxidation marker levels of goat meat stored (a) at 26°C for 6 h and (b) at −18°C for 30 days. Values presented are the mean 2-thiobarbituric acid reactive substances values and standard error bars. ^a,b^ Within each storage temperature, different superscripts indicate differences between dietary treatments (p≤0.05) tested using LSD Fisher test (p<0.05).

On the other hand, TBARS values of meat stored at 26°C for 6 h are shown in Figure 2a. It is observed that the goat meat of the control group was significantly more oxidized than the meat sample of the LD and AA treatments, without significant differences between them but different with control. The MDA values were 4.59, 1.72, and 1.08 mg MDA/kg of meat for the control, LD and AA groups, respectively.

Finally, the TBARS values for goat meat stored at −18°C for 30 d are shown in Figure 2b. It is observed that meat MDA values of the control group were significantly higher (3.80 mg MDA/kg of meat) than the values obtained in the samples of the LD and AA treatments (1.30 and 1.55 mg MDA/kg of meat, respectively), with no difference between them.

It has been reported that the incorporation into the goat diet of *Moringa oleifera* leaves, a plant species with a high polyphenol content cultivated in Asia, improves the oxidative stability of the meat [33]. Nevertheless, it has been reported that supplementations with pine bark did not have significant effects on the TBARS values in goat meat [12]. The same way, other studies [19] showed that the inclusion of quebracho tannins in the lamb diet improves the color stability of the meat, but not its oxidative stability. Therefore, our results are in line with several studies that reported improved oxidative stability of lamb meat in response to the administration of diets rich in phenolic compounds, saponins and essential oils [34].

To our knowledge, this is the first report showing the pos itive effect of the incorporation in the diet of goats of woody species LD and AA in oxidative stability of the meat. The protective effect of LD and AA diets against meat lipid peroxidation found in the present work cannot be ascribed to the presence in meat of antioxidant compounds as their contents and antioxidant activity were not significantly different with control. Thus, the observed increase in oxidative stability should be based on the variation of the susceptibility of the meat lipids to oxidation due to a possible change in their composition.

### Fatty acid composition in goat meat

Sixteen fatty acids were identified and quantified in the fat extract of the meat from goats supplemented with LD and AA leaves, which consisted of nine saturated fatty acids (SFA: C6: 0, C10: 0, C14: 0, C15: 0, C16: 0, C17: 0, C18: 0, C20: 0, and C21: 0), four monounsaturated fatty acids (MUFA: C15: 1, C16: 1, C18: 1n9t, C18: 1n9c) and three PUFA (C18: 3n6, C18: 2n6c, and C20: 2) (Table 4). The major ones are oleic, palmitic and estearic acids, C18: 1n9c, C16: 0, and C18: 0, respectively. Fatty acid general profile and MUFA/SFA or PUFA/SFA ratios were similar among treatments. However, slight variations in particular cases were observed; C18: 1n9c content of LD treatment presented significant differences with control or AA treatment. With respect to pentadecanoic acid (C15:0) and *cis*-10-pentadecenoic acid (C15:1) in muscle AA treatment had significant differences (p<0.05) with the control; being higher in saturated but lower in the unsaturated fatty acids. When woody species were added to the forage, the C18: 2n6c concentration in meat increased by 36% in comparison with goats fed with forage without woody species. The supplementation with woody species did not produce significant differences among SFA levels in contrast to MUFA contents in meat (p = 0.007). The concentration of PUFA was numerically higher in meat of goats supplemented with tannin rich leaves; although, no statistical differences were found.

The proportions of PUFA/SFA in the present study were lower than the recommended ratio of 0.45 to balance the negative effects of dietary saturated fat on human health [1]; being for supplemented animals 0.13 for goat fed with LD and 0.15 for goats fed with AA. These results showed an increase in the PUFA/SFA ratio with respect to the control group goats.

Previous studies have investigated possible feeding strat egies to increase PUFA content in meat and milk, through the manipulation of ruminal biohydrogenation. Min et al [26] reported that CT had an inhibitory effect on ruminal microorganisms. *In vitro* studies showed that the incubation of ruminal fluid with CT reduced the biohydrogenation of linoleic acid [11] and linolenic acid [35], indicating lower activity of ruminal microorganisms by the presence of tannins. Lee et al [12] studied the chemical composition and quality characteristics of meat of goats fed with wood-derived CT as pine bark (13% CT on DM) compared to bermudagrass hay. They did not find significant differences in meat lipid profile, PUFA/SFA, and meat traits attributed to dietary CT from pine bark. In the same trend, in our study, we found that PUFA were numerically higher and SFA were lower in meat of goats supplemented with native woody species as a source of tannins, although, no significant differences were found. This is also in good agreement with the Vasta et al [36], report concerning meat characteristics of lambs supplemented with commercial quebracho tannins in their diet. In our study we observed that the incorporation of woody species in goat diets as a source of CTs improved the oxidative stability of meat during storage, indicating that this antioxidant potential of the meat could be due to the effect on lipid composition. In this case, the presence of CT in the diet could play an important role in the ruminal metabolism reducing biohydrogenation and exerting positive effects on the oxidative stability of the meat.

The higher TPC the lower oxidation level of the meat, suggest ing that these species are an important source of polyphenols, giving caprine meat a greater resistance to oxidation compared to polyphenol free diets. Generally, the present study has demonstrated that LD and AA may be exploited as dietary supplement to improve oxidative stability of meat from goat livestock.

## CONCLUSION

Under the experimental conditions of this study, feeding antioxidant-rich native species had no significant effect in terms of animal growth or yield, neither in the meat quality parameters. The most important finding of this experiment was that the inclusion of woody species in the diet of goats promoted not only a lower oxidation status of the meat after slaughter but also increased the resistance of the meat to lipid oxidation under refrigerated or frozen storage. Results also indicated that this enhancement was not due to the increase of the TPC concentration or antioxidant activity in the muscle, but it was probably due to the modulation of the ruminal biohydrogenation producing changes in meat’s lipid profile. The inclusion of AA and LD native species in goat diet enhanced meat oxidative stability.

## Figures and Tables

**Table 1:** Ingredients and chemical composition of the experimental diet

Items	Control	*Larrea divaricata*	*Acacia aroma*
Ingredients (g/kg as fed)			
Alfalfa hay	510	450	400
Corn	270	225	295
Soybean meal	220	200	180
*Larrea divaricata* leaves	-	125	-
*Acacia aroma* leaves	-	-	125
Chemical composition (% on dry matter)			
Crude protein	16.7	16.7	16.4
Neutral detergent fiber	42.2	41.1	40.1
Crude fat	3.2	3.2	3.2
Total phenols1)	0.38	12.69	5.47
Metabolizable energy2) (Mcal/kg MS)	2.6	2.5	2.5

1) Expressed as g of gallic acid equivalents per 100 g of dry matter (GAE/100 g DM).

2) Calculated from a Composition Table of Forages according to NRC [14] report.

**Table 2:** Effects of tannin-rich leaves in diets on growth performance and carcass traits of goats

Items	Control	*Larrea divaricata*	*Acacia aroma*	SEM	p-value
Slaughter weight (kg)	13.06	12.92	14.02	0.87	0.67
Hot carcass weight (kg)	5.60	5.58	5.65	0.41	0.99
Cold carcass weight (kg)	5.44	5.52	5.61	0.43	0.96
Total DMI (g/d/kg BW^0.75^)	67.8	67.0	65.5	2.7	0.83
ADG (g/d)	65	55	77	15	0.62
Carcass measurements and indexes					
Internal carcass length (cm)	46.75	45.33	46.58	1.21	0.67
Leg length (cm)	28.08	29.00	30.75	0.75	0.07
Croup perimeter (cm)	40.25	39.67	40.33	1.41	0.95
Chest depth (cm)	20.00	19.42	19.75	0.63	0.81
Chest width (cm)	9.42	9.08	8.50	0.49	0.43
Croup width (cm)	11.83	10.58	10.5	0.64	0.28
Carcass compactness index (kg/m)	116.13	121.38	119.83	7.04	0.85
Leg compactness index (kg/m)	0.42	0.36	0.34	0.02	0.08
Subcutaneous fat (%)	1.50	1.50	1.42	0.16	0.91
KKCF (%)	0.50	0.33	0.25	0.08	0.12

SEM, standard error of mean (n = 6); DMI, dry matter intake; BW, body weight; ADG, average daily gain; KKCF, Kidney knobb carcass fat.

**Table 3:** Effect of the dietary tannin-rich leaves on meat quality of feeding goats

Item	Control	*Larrea divaricata*	*Acacia aroma*	SEM	p-value
Meat pH	5.58	5.49	5.52	0.006	0.53
Water retention capacity (%)	38	34.83	43.83	6.31	0.18
Color parameters1)					
L* values	55.9	55.78	56.42	1.04	0.9
a* values	9.50	9.24	8.83	0.49	0.61
b* values	1.55	2.04	2.06	0.23	0.2
Hue angle	6.75	5.15	4.75	0.86	0.22
Chroma	11.05	11.29	10.89	0.60	0.9
Antioxidant activity2) (mg of GAE/g of meat)	14.20	15.04	15.51	0.92	0.57
Total phenolic content (mg of GAE/g of meat)	10.7	10.9	11.1	0.07	0.85

SEM, standard errors of mean (n = 6); GAE, gallic acid equivalents.

1) Color development after meat aging and blooming.

2) Radical-scavenging capacity against 2,2-diphenyl-1-picrylhydrazyl radical (DPPH·).

**Table 4:** Fatty acid composition in meat intramuscular fat of goats (n = 6) fed *Larrea divaricata* and *Acacia aroma* supplemented diets

Fatty acids	Control	*Larrea divaricata*	*Acacia aroma*	SEM	p-value
C6:0	3.46	2.09	2.85	0.83	0.49
C10:0	1.78	1.36	1.64	0.24	0.43
C14:0	2.78	2.46	2.68	0.26	0.66
C15:0	0.36	0.74	0.98	0.19	0.07
C16:0	23.83	22.82	22.72	0.84	0.82
C17:0	1.55	1.63	1.1	0.24	0.27
C18:0	14.67	14.61	14.99	0.82	0.97
C20:0	2.05	1.27	1.4	0.67	0.65
C21:0	1.29	1.39	1.89	0.2	0.14
C15:1	1.97^a^	1.62^ab^	1.26b	0.15	0.02
C16:1	1.4	1.95	1.39	0.44	0.69
C18:1n9t	1.85^b^	1.81^b^	1.25^a^	0.16	0.03
C18:1n9c	41.44^b^	33.67^a^	39.19^b^	1.36	0.002
C18:2n6c	2.53	3.45	4.97	0.89	0.16
C18:3n6	1.93	2.64	2.08	0.63	0.69
C20:2	0.87	0.50	0.74	0.25	0.56
SFA	51.32	48.37	50.36	1.82	0.47
MUFA	46.66^b^	39.05^a^	42.99^ab^	1.5	0.007
PUFA	5.33	6.59	7.79	1.24	0.42
MUFA/SFA	0.92	0.81	0.85	0.05	0.21
PUFA/SFA	0.1	0.13	0.15	0.02	0.35

SEM, standard error of mean (n = 6); SFA, saturated fatty acids, C6:0–21:0; MUFA, monounsaturated fatty acids, C15:1, C16:1, C18:1n9t, C18:1n9c; PUFA, polyunsaturated fatty acids, C18:3n6, C18:2n6c, C20:2.

ab Different superscripts indicate differences among treatments (p<0.05).
